# Long- and short-term complications of episiotomy

**DOI:** 10.4274/tjod.00087

**Published:** 2016-09-15

**Authors:** İsmet Gün, Bülent Doğan, Özkan Özdamar

**Affiliations:** 1 Gülhane Military Medical Academy, Haydarpaşa Training and Research Hospital, Clinic of Obstetrics and Gynecology, İstanbul, Turkey; 2 İstanbul Medeniyet University Faculty of Medicine, Department of Obstetrics and Gynecology, İstanbul, Turkey

**Keywords:** Episiotomy, urinary incontinence, anal incontinence, Sexual dysfunction

## Abstract

Although extensively applied in obstetrics practice to facilitate delivery by increasing the vaginal birth conduit, most episiotomy studies are in the context of short- or medium-term outcomes, and the number of studies investigating the long-term effects is insufficient. Episiotomy is often considered associated with urinary and/or anal incontinence and dyspareunia; however, there is no concrete evidence for this issue. Current meta-analyses and reviews that assessed the studies available in the literature revealed that episiotomy does not decrease the rates of urinary incontinence, perineal pain, and sexual dysfunction and that routine episiotomy does not prevent pelvic floor damage; thus, the recommended use of mediolateral episiotomy is restricted, rather than routine. According to the limited number of studies on sexual function, there seems to be a linear relationship between the degree of perineal laceration and postpartum dyspareunia. It is still not clear whether episiotomy has any impact on pelvic floor relaxation, pelvic organ prolapse, and sexual dysfunction in the long term.

## PRECIS:

The long-term influences of episiotomy on urinary and/or fecal incontinence, pelvic floor dysfunction, sexual function, and dyspareunea are still not clear and studies on these issues are necessary.

## INTRODUCTION

For a succesful vaginal birth, vaginal and cervical expansion should occur slowly and the tissue should be allowed to stretch in a proper manner. At this time, spontaneous tears may ensue in rapid descent, particularly during the fetal head descent and the formation of vaginal dilatation. Even if these tears, as described by Fernando who divided them into four degrees, most frequently involve perineal skin and mucosa (1^st^ degree), they may extend to perineal muscle (2^nd^ degree), anal sphinchter complex (3^rd^ degree), and anal mucosa (4^th^ degree). Another reason for vaginal tears at birth is controlled and properly made perineal incisions performed at the end of the second stage of labor to ease parturition by increasing the vaginal diameter, known as episiotomy^(1)^. Two types have been described; median (from the posterior fourchette to the anus) and mediolateral (from hymenal ring downwards with at least a 45-degree angle). However, standardization in the method of application and repair of episiotomy is still lacking today. Additionally, in the majority of studies conducted on this issue to date, the parameters likely to influence the healing process and long-term outcomes are not clear.

## EARLY STUDIES AND SHORT-TERM EFFECTS

After being described by Ould^(2)^ in 1741, episiotomy was first recommended to be applied in a mediolateral fashion in all births of nulliparous women in order to protect the fetal head from trauma and the pelvic floor from extreme lacerations in 1921^(3)^. For years, episiotomy was believed to be repaired more easily and to reduce the risk of severe lacerations in the short term, and to protect against pelvic floor relaxation, sexual dysfunction, and long-term urinary and/or fecal incontinence, as compared with spontaneous vaginal or perineal tears. It was also considered associated with neonatal benefits such as lower incidences of asphyxia, cranial trauma, cerebral hemorrhage, mental retardation, and shoulder dystocia. Consequently, episiotomy was used extensively until the first half of the nineteenth century and the frequency of application gradually increased. However, in the second half of the twentieth century, increasing evidence that episiotomy did not actually provide these benefits began to be published^(4)^. Thereupon, Thacker and Banta^(4^) reviewed the related studies conducted between 1860 and 1980 and analyzed the results to investigate whether episiotomy provided any benefits. As a result, they reported that the number of studies available was insufficient and that the studies were not reliable enough to substantiate their hypotheses; thus, the results did not support the routine use of episiotomy. Moreover, it was indicated that postpartum pain and discomfort became evident and serious complications, and maternal death might even occur after episiotomy^(4)^. During the defined period and the following 10 years, publications mostly originated from the United States and were of minor scale, in addition, as almost all dealt with midline episiotomy. There is a very limited number of publications on mediolateral episiotomy and the most comprehensive study was from Argentina, a randomized controlled trial that included 2.606 women from 8 centers^(5)^. In that study, routine, restrictive or selective episitotomies were compared and episiotomy rates were reported to be 82.6% and 30.1% in the restrictive and routine groups, respectively. Perineal lacerations of 3^rd^ or 4^th^ degree were reported lower in the restrictive group than in the routine group (1.2% vs. 1.5%). Subsequently, the review by Homsi et al.^(6)^ indicated the possible drawbacks of routine episiotomy to be the extension of the episiotomy incision, unsuitable anatomic outcomes, increased blood loss and hematoma formation, pain, inflammation, infection and dehiscence within the episiotomy region, sexual dysfunction, and increased costs (Table 1).

## EPISIOTOMY AND LONG-TERM EFFECTS

A significant proportion of the studies relevant to episiotomy actually assessed short- and medium-term period outcomes; long-term complications associated with episiotomies were not explicit. In a systematic review published in 2005 in the Journal of the American Medical Association that included only 26 prospective randomized controlled trials even though 986 publications had been published between 1950 and 2004, data beyond the postpartum first year was only provided in two studies,^(7)^ one of which was conducted by Sleep and Grant^(8)^ in 1987 who compared routine versus restrictive episiotomy during spontaneous vaginal delivery within a single center. That study, which was initiated with 1.000 women in 1984, was terminated with 674 women in the 3^rd^ postpartum year and reported that there was no significant difference between the two groups in terms of dyspareunia and urinary incontinence. The other study was conducted by Rockner^(9)^ in 1990 and reported no statistically significant difference in the incidence of urinary incontinence between groups with and without episiotomy at the postpartum 4^th^ year. However, no study has provided data on anal incontinence beyond the 1^st^ postpartum year. As a result, there is no evidence to support the maternal benefits of routine episiotomy.

One of the largest studies on the long-term complications of episiotomy was conducted in France with the participation of two hospitals that adopted diverse policies regarding episiotomy^(10)^. Long-term outcomes of restrictive and routine episiotomy were compared in this study, including deliveries of 774 nulliparous women with singleton and cephalic presentation pregnancies between 37 and 41 gestational weeks. Four postpartum years later, 627 women responded with a 81% return rate and the patient distribution was 320 versus 307 women; the episiotomy rates were 49% and 88% in the restrictive and routine episiotomy groups, respectively. The rates of urinary incontinence, perineal pain, and dyspareuneu were lower in the restrictive group than in the routine episiotomy group with rates of 26% vs. 32%, 6% vs. 8%, and 18% vs. 21%, respectively, but not there was no significant difference. Similarly, fecal and flatus incontinences were lower in the restrictive group than in the routine episiotomy group with rates of 11% vs. 16%, and 8% and 13%, respectively; statistical significance was reached only for flatus incontinence. Consequently, the authors stressed that routine episiotomy did not protect against anal and urinary incontinence, there was even a increased risk of anal incontinence in the long term, and that restrictive episiotomy should be preferred to routine episiotomy.

In the early 2000s, publications reporting the increasing incidence of severe obstetric lacerations began to emerge and in the United Kingdom, and the incidence of perineal lacerations of grade 3 or 4, with a reported incidence of 1.8% in 2000, was reported to rise to 5.9% in 2011, which exhibited a 3-fold increase^(11)^. An increased risk for severe perineal lacerations were indicated associate with increased maternal age, instrumental delivery, Asian race, higher socio-economic status, birth weight of 4.000 g or above, and shoulder dystocia. Some publications reported that selective episiotomy decreased the likelihood of 3^rd^ or 4^th^ degree perineal lacerations;^(12)^ whereas, in a large observational study that included approximately 3.000 births, risk of perineal lacerations was reported associated with a set of factors, mainly including median episiotomy^(13)^. Today, there are two remarkable retrospective studies regarding mediolateral episiotomy. The first is a retrospective population-based study conducted in 2001, which comprised 284.000 vaginal births^(14)^. In that study, risk factors for 3^rd^ degree perineal tears were investigated and episiotomy rate was reported as 35.1%, the rate of 3^rd^ degree perineal tears was presented much lower than those in previous reports (1.94% vs. 4-5%). The authors concluded that forceps delivery was the most remarkable risk factor for 3^rd^ degree perineal laerations [odds rate (OR), 3.33; 95% confidence Interval (CI): (2.97-3.74)] and that mediolateral episiotomy should be performed as a primary measure in case of fetal macrosomia to prevent 3^rd^ degree perineal lacerations. The latter was a retrospective study conducted by Baumann et al.^(15)^ in 2006 that included 40.000 vaginal births. In contrast to the previous study, the rate of anal sphincter laceration in primiparous women was reported as high as 5.2% and 17 obstetric risk factors, which may result in sphincter injury. Moreover, it was emphasized that anal sphincter laceration was most strongly associated with episiotomy [OR, 3.23; 95% CI: (2.73-3.80)] and forceps delivery [OR, 2.68; 95% CI: (2.17-3.33)].

Beyond the causes of severe obstetric lacerations, there were no concrete data on repair and long-term outcomes. In a retrospective case-control survey study, 171 women who underwent anal sphincter rupture surgery between 1971 and 1990 were matched with 171 control women for time and number of deliveries and all women were interrogated twice in both 1996 and 2005 as to whether there had been any increase in sexual and anorectal symptoms, regardless of the menopausal state; a statistically significant increase was determined in study group^(16)^. In particular, the rates of anorectal symptoms in 1996, when questioned for the first time, were 16% and 38% in control and study groups, respectively, whereas in 2005, they were 22% and 61%, respectively, which revealed that the increase in the variation of rates, as the years advanced, was statistically significant in the study group (p<0.0001). Additionally, in the questionnaire in 2005, dyspareunia and fecal incontinence during intercourse were investigated and found significantly different between the controls and study patients (13% vs. 29%, p=0.01 and 1% vs. 13%, p=0.05, respectively). Similar results were reported in another study conducted in 1988; the anal incontinence rate in women with complete perineal rupture occuring at vaginal delivery, as declared after a mean of 78 months was 22%, whereas it was 0% in the control women (p<0.01)^(17)^. Even though perineal laceration was succesfully repaired, Poen et al.^(18)^ also affirmed the anal incontinence rate after 5 years to be 40%.

## RESTRICTED INSTEAD OF ROUTINE EPISIOTOMY?

The first Cochrane review available in the literature, in the context of benefits and possible risks of routine episiotomy, which aimed to compare routine versus restricted episiotomy as well as midline versus mediolateral episiotomy, was published in 1999, and revised in 2004 and 2009^(19)^. The authors included only 8 randomized controlled trials, comprising a total of 5.541 women, because most of the studies were of low-quality^(5,20,21,22,23,24,25,26)^. The frequency episiotomy was 75.15% in the routine group, and 28.40% in restrictive group. The limitations of the review were the limited data for episiotomy technique and lack of high-quality studies included in the review, although there were 3 studies available comparing midline and mediolateral episiotomies. Based on the results of the review, the incidence of any anterior trauma was significantly higher in the restrictive group than in the routine group [relative risk (RR), 1.84; 95% CI: (1.61-2.10)]. However, the only data on long-term episiotomy complications available was dyspareunia at 3 postpartum years and there was no significant difference between the groups [RR, 1.21; 95% CI: [0.84-1.75)]. Consequently, it was reported that routine episiotomy did not reduce the rates of urinary incontinence, pain, and sexual dysfunction, and that it has no benefit to the newborn. The recommendations of both National Institute of Clinical Excellence and Royal College of Obstetricians and Gynaecologists are similar and compatible with each other. In 2006, the American College of Obstetricians and Gynecologists stated that the frequency of anal sphincter and rectal mucosa injury in vaginal deliveries with median episiotomy was higher than in those with mediolateral episiotomy and they recommended restrictive mediolateral episiotomy, if necessary (Level A), and also expressed that routine episiotomy did not prevent pelvic floor damage (Level B)^(27)^.

Prophylactic episiotomy still continues to be widely used today, although the number of publications that recommend against its routine use is higher. Obstetricians’ perception that episiotomy decreases the risk of perineal trauma as compared with spontaneous tears, apparently without having any basis of scientific evidence, constitutes the most substantial justification for this practice^(28)^. The implementation of episiotomy is likely to be influenced by the physician’s working environment, conditions and individual diversities as well as mother and fetal factors. One study reported that midwives were more prone to perform episiotomies than physicians,^(29)^ and another indicated that faculty providers performed episiotomies at a lower rate than private providers^(30)^. The study by Gossett and Dunsmoor Su^(31)^ revealed individual differences more clearly.

## EPISIOTOMY AND SEXUAL DYSFUNCTION

Postpartum sexual life has recently been a subject of research. Studies have also demonstrated that postpartum sexual problems are common in the short term. Although perineal pain and dyspareunia that occur in the postpartum period are considered the main issues that prevent normal sexual activity, our knowledge on this issue is lacking because there are insufficient studies comparing ante- and postpartum sexual activity. According to the results of the study conducted by Abdool et al.^(32)^ in 2009, perineal pain and dyspareunia results from perineal trauma, lacerations, episiotomy and forceps or vacuum use at delivery. Moreover, the authors also reported that perineal pain develops in 42% of patients in the early postpartum period and declines to 22% and 8% in the postpartum 8^th^ and 12^th^ weeks, respectively. Another study that included 921 primiparous women stated that 25% of women had lower sexual desire and functioning at the postpartum 6^th^ month and 42% and 22% of women had dyspareunia at the 3^rd^ and 6^th^ postpartum months, respectively^(33)^. In the same investigation, it was reported that women with a second degree perineal trauma had 80% more dyspareunia symptoms, and those who had third degree perineal trauma had 270%, as compared with women who had no perineal trauma. However, there is very limited high-level evidence regarding long-term postpartum sexual dysfunctions. A limited number of studies that compared routine and restrictive episiotomy outcomes reported that the frequency of dyspareunia at the 3^rd^ and 4^th^ postpartum years did not differ between the groups^(8,10,19)^. A study from the Netherlands stressed that dyspareunia was significantly more common in women who underwent repair surgery for anal sphincter rupture than in those who did not, when the patients were questioned 15 years after their delivery (dyspareunia 13% vs. 29%, respectively, p=0.01)^(16)^.

## CONCLUSION

Even though a substantial number of publications do not recommend the implementation of routine prophylactic episiotomy, it still continues to be widely performed. It is not clear as to which approach should be adopted in operative delivery; however, the hitherto gathered data supports restrictive rather than routine episiotomy. Moreover, data as to whether routine episiotomy reduces the incidence of severe obstetric lacerations is lacking, as well as whether episiotomy improves the long-term risks of pelvic floor relaxation, pelvic organ prolapse, urinary incontinence, and dyspareunia remains unclear, and further studies on this issue are still warranted.

## Figures and Tables

**Table 1:**
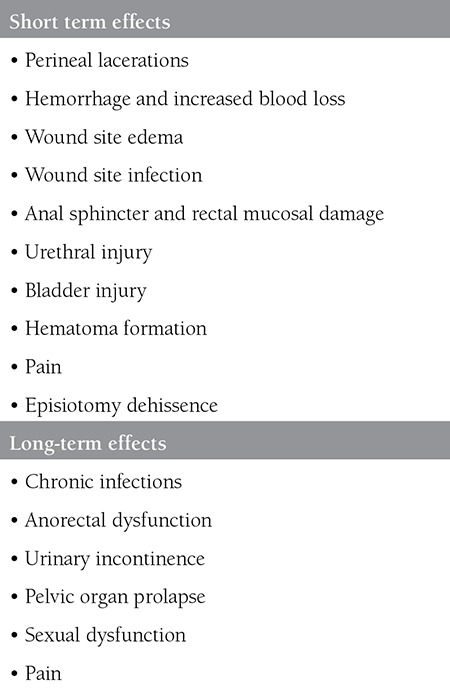
Short and long-term consequences of performing an episiotomy
